# A bibliometric analysis of artificial intelligence applications in macular edema: exploring research hotspots and Frontiers

**DOI:** 10.3389/fcell.2023.1174936

**Published:** 2023-05-15

**Authors:** Haiwen Feng, Jiaqi Chen, Zhichang Zhang, Yan Lou, Shaochong Zhang, Weihua Yang

**Affiliations:** ^1^ Department of Software Engineering, School of Software, Shenyang University of Technology, Shenyang, Liaoning, China; ^2^ Department of Computer, School of Intelligent Medicine, China Medical University, Shenyang, Liaoning, China; ^3^ Shenzhen Eye Institute, Shenzhen Eye Hospital, Jinan University, Shenzhen, China

**Keywords:** bibliometric analysis, deep learning, artificial intelligence, macular edema, ophthalmology, machine learning

## Abstract

**Background:** Artificial intelligence (AI) is used in ophthalmological disease screening and diagnostics, medical image diagnostics, and predicting late-disease progression rates. We reviewed all AI publications associated with macular edema (ME) research Between 2011 and 2022 and performed modeling, quantitative, and qualitative investigations.

**Methods:** On 1st February 2023, we screened the Web of Science Core Collection for AI applications related to ME, from which 297 studies were identified and analyzed (2011–2022). We collected information on: publications, institutions, country/region, keywords, journal name, references, and research hotspots. Literature clustering networks and Frontier knowledge bases were investigated using bibliometrix-BiblioShiny, VOSviewer, and CiteSpace bibliometric platforms. We used the R “bibliometrix” package to synopsize our observations, enumerate keywords, visualize collaboration networks between countries/regions, and generate a topic trends plot. VOSviewer was used to examine cooperation between institutions and identify citation relationships between journals. We used CiteSpace to identify clustering keywords over the timeline and identify keywords with the strongest citation bursts.

**Results:** In total, 47 countries published AI studies related to ME; the United States had the highest H-index, thus the greatest influence. China and the United States cooperated most closely between all countries. Also, 613 institutions generated publications - the Medical University of Vienna had the highest number of studies. This publication record and H-index meant the university was the most influential in the ME field. Reference clusters were also categorized into 10 headings: retinal Optical Coherence Tomography (OCT) fluid detection, convolutional network models, deep learning (DL)-based single-shot predictions, retinal vascular disease, diabetic retinopathy (DR), convolutional neural networks (CNNs), automated macular pathology diagnosis, dry age-related macular degeneration (DARMD), class weight, and advanced DL architecture systems. Frontier keywords were represented by diabetic macular edema (DME) (2021–2022).

**Conclusion:** Our review of the AI-related ME literature was comprehensive, systematic, and objective, and identified future trends and current hotspots. With increased DL outputs, the ME research focus has gradually shifted from manual ME examinations to automatic ME detection and associated symptoms. In this review, we present a comprehensive and dynamic overview of AI in ME and identify future research areas.

## 1 Introduction

Macular edema (ME) is a common, critical disease caused by retinal vein occlusion, diabetic retinopathy (DR), chronic uveitis, and eye injury, of which, macular lesions are the leading cause of disease. Clinically significant ME is manifested by retinal thickening which impacts the macula center, is defined by central retinal thickness >250–300 μm, and examined using Optical Coherence Tomography (OCT) (Hee et al., 1995). ME also involves fluid accumulation in retinal layers which is a common morphological manifestation in different retinal diseases (Daruich et al., 2018). Therefore, it is vital to quantitatively analyze ME research areas, the disease *status quo*, and future prospects related to disease progression.

Bibliometrics is used to analyze different knowledge carriers using mathematics and statistics (Cancino et al., 2017). It evaluates development trends in target disciplines/scientific fields by analyzing database and document characteristics to identify research hotspots and key research directions. In recent years, bibliometric analysis have been successfully used in orthopedics, ophthalmology, and gynecology (Qiu et al., 2018; Huang et al., 2020; He J. et al., 2021). Additionally, the approach is invaluable for writing guidelines, making clinical decisions, and importantly, treating different diseases. However, bibliometric analyses related to ME in ophthalmology remains under-studied (Narotsky et al., 2012; Seriwala et al., 2015; Khan et al., 2016), therefore, we systematically investigated this research area to characterize the *status quo* and identify research hotspots.

## 2 Materials and methods

**FIGURE 1:**
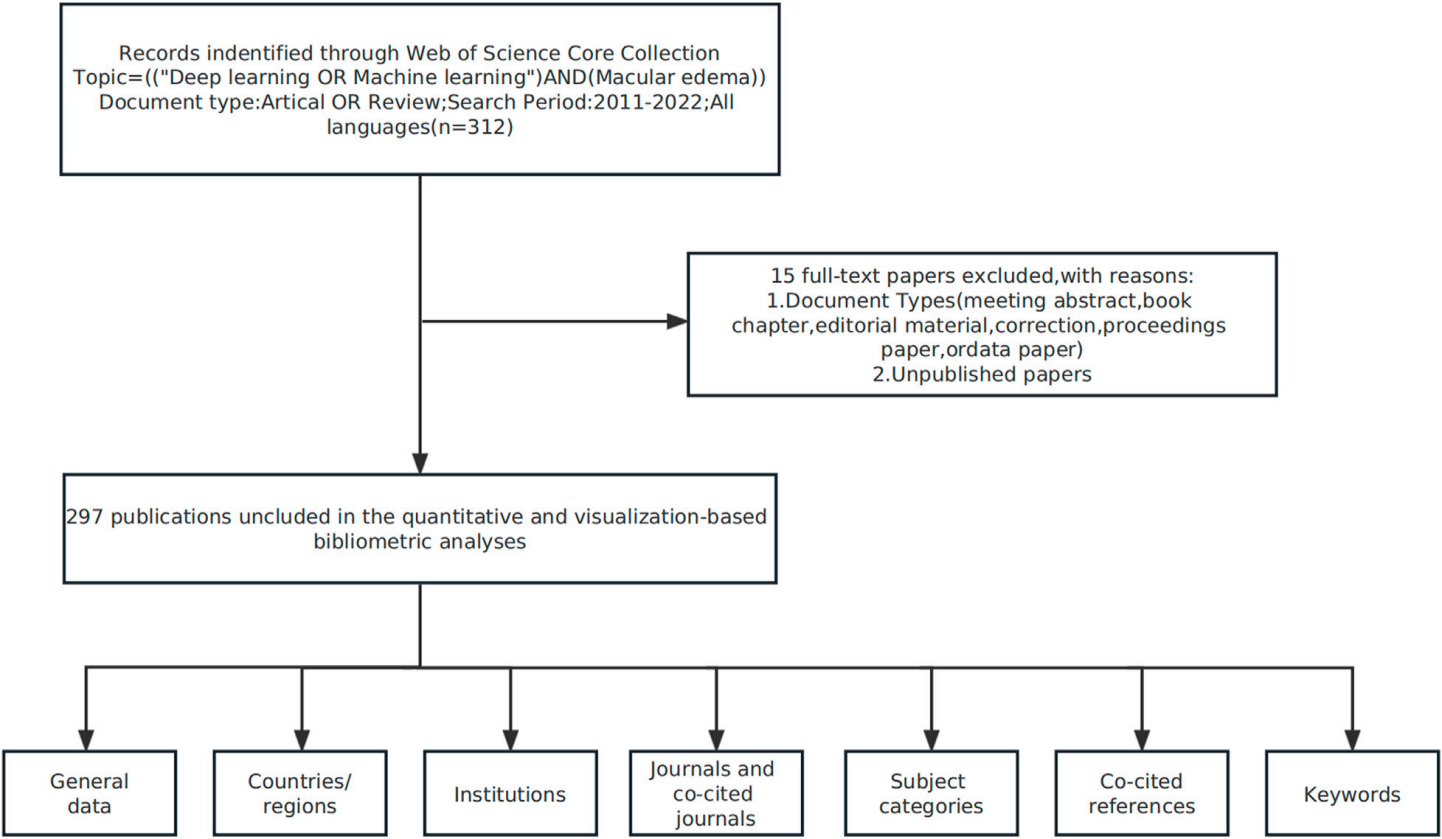
Study flow chart showing bibliometric analyses and selection criteria of macular edema studies.

On 1st February 2023, we downloaded data from the Web of Science Core Collection (2011–2022) using: “machine learning” OR “deep learning” OR “convolutional neural network*” OR “CNN*” OR “Recurrent neural network*” OR “RNN” OR “Fully Convolutional Network*” OR “FCN*” search terms. The parallel search subject was ME and relevant studies included basic information on: authors, abstracts, keywords, titles, institutions, journals, countries/regions, and references. Indexed database studies were included, but meeting abstracts, book chapters, data papers, proceedings, editorials, and repeat articles, and unpublished studies containing limited data were excluded. A summary of this process is shown (Figure 1).

We also examined publication characteristics: keywords, institutions, countries/regions, and journals. We also used the H-index which evaluates the scientific value of research and measures author/journal scientific productivity (Eyre-Walker and Stoletzki, 2013). To represent collaborative networks across journals/institutes/countries/keywords and facilitate co-occurrence investigations, we used R language bibliometrics software (Massimo Aria and Corrado Cuccurullo), CiteSpace (Drexel University, PA, United States), and VOSviewer (Leiden University, Holland). The R language bibliometric package is widely used in statistical computation and graphics (Aria and Cuccurullo, 2017) and was used to extract the top 10 keywords and cluster them into themes/evolution/hierarchical clustering/topic trends. From collaborative data, we used the VOSviewer to provide a comprehensive and detailed view of bibliometric maps. We also generated a cooperation relationship diagram between institutions and also a reference relationship diagram between foresight to analyze cooperation outputs between institutions and reference relationships between disciplines. CiteSpace was used to investigate knowledge from the literature and visualize data (Chen, 2004). We also generated knowledge maps, performed discipline evolution analyses, and determined burst keywords (BKs) to identify recurrent keywords.

## 3 Results

### 3.1 Study distribution (year of publication)

**FIGURE 2:**
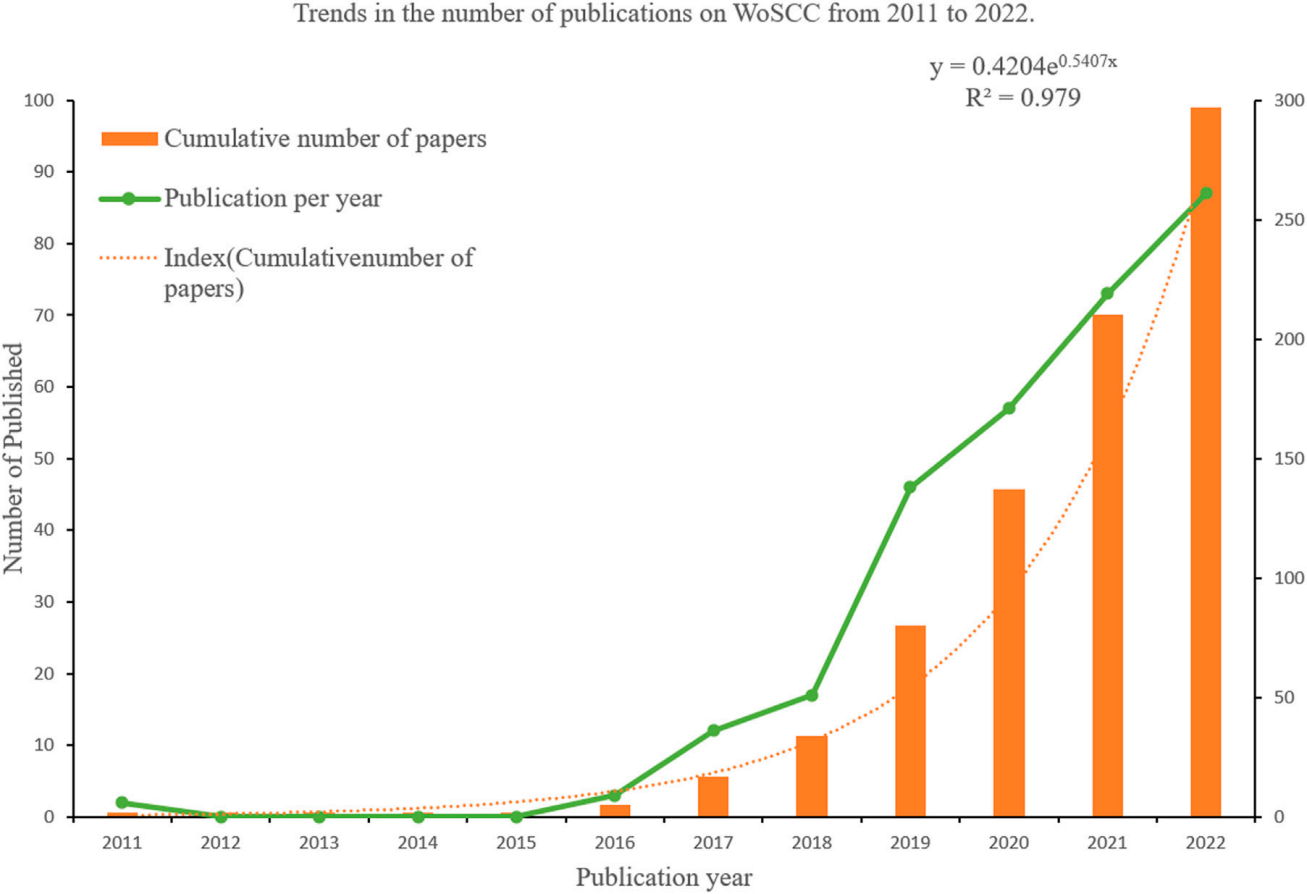
Macular edema publications; publication trends between 2011 and 2022 (publication years).

We observed that AI in ME research commenced in 2011. From 2011 to 2022, we identified 297 papers and identified AI-associated ME publication trends (Figure 2). While this type of research emerged in 2011, it fell silent from 2012 to 2015. However, from 2016, in-depth learning approaches combined with ophthalmology led to increased ME research outputs, and paper outputs increased year on year suggesting an important research trend had been established.

### 3.2 Institutes/countries/regions

**FIGURE 3:**
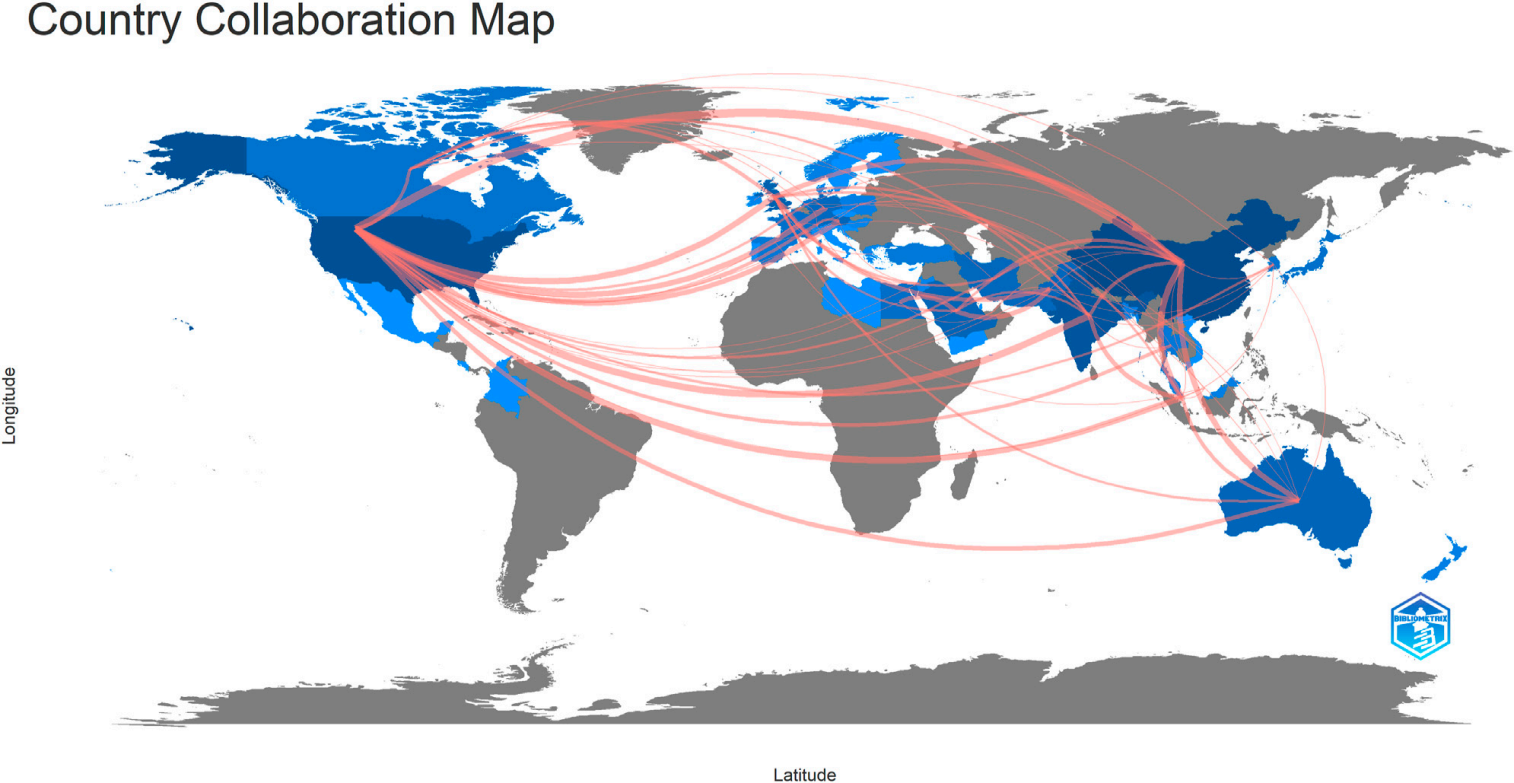
Collaboration map showing how countries/regions contributed to/collaborated on macular edema publications.

**FIGURE 4:**
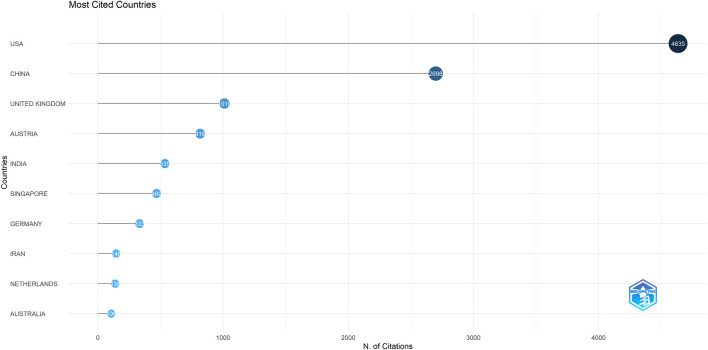
Diagram showing how the most cited countries/regions contributed to/collaborated on macular edema publications.

Forty-seven countries/regions published ME studies - the top 10 countries (Table 1) and collaborations (Figures 3, 4) are indicated. China published the most studies (101), then the United States (73), India (48), and the United Kingdom (23). Some countries (United States, China, and the United Kingdom) showed high centrality (dark blue—Figure 3) suggesting important regional roles in/contributions to ME research. H index is a mixed quantitative index, which can be used to evaluate the quantity and level of academic output of a country or institution. Because the United States has the highest H-index, it has the greatest influence in the field of macular edema.

**TABLE 1:** Top ten institutions and countries/regions.

Rank	Countries/Regions	Count	Citations	H-index	Institutions	Count	H-index
1	China	101	3,286	23	Medical University Of Vienna	15	11
2	United States	73	7,302	28	National University Of Singapore	12	10
3	India	48	4,026	14	University Of California System	12	7
4	England	23	1,419	10	Singapore National Eye Center	11	9
5	Singapore	21	718	11	Shanghal Jiao Tong University	10	6
6	Saudi Arabia	18	157	7	Egyptian Knowledge Bank EKB	9	3
7	Australia	17	613	8	Indian Institute Of Technology System IIT System	9	7
8	Austria	17	1,011	11	Isfahan University Medical Science	9	5
9	Pakistan	15	157	8	Shantou University	9	4
10	Iran	14	349	7	Cleveland Clinic Foundation	8	6

**FIGURE 5:**
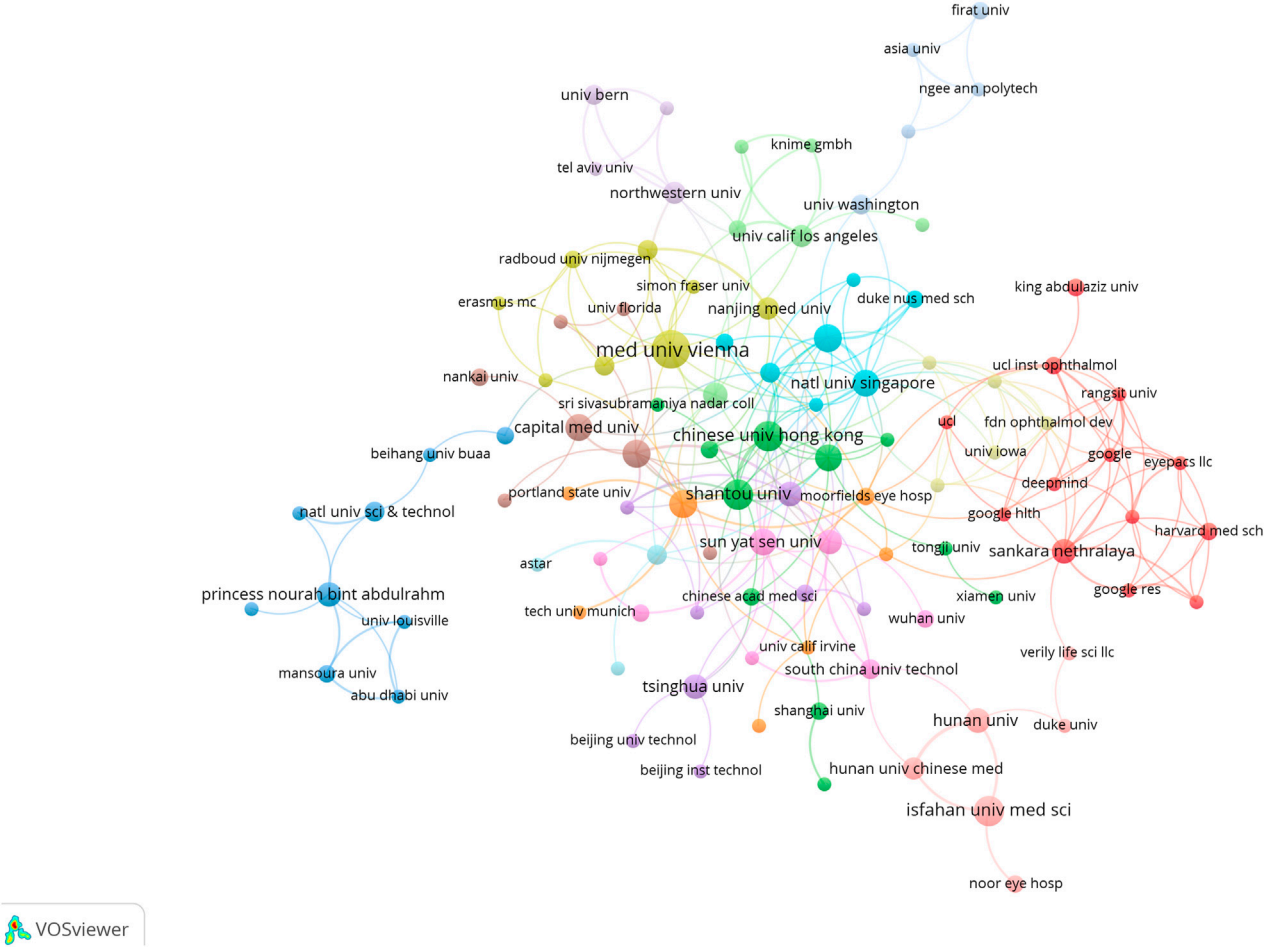
Institutional cooperation/contributions to publications.

In total, 613 institutes generated ME publications; the top ten are shown (Table 1). Institutions are also outlined (Figure 5). The Medical University of Vienna had the most publications (15), followed by the National University of Singapore (12), the University of California (12), and the Singapore National Eye Center (11).

We also cataloged research institutions with outstanding contributions to the ME field (Figure 5) using VOSviewer. Regional institutional distributions showed distinct aggregation effects, indicating that academic research was concentrated to a few countries. From a literature perspective, most institutions were based in universities and scientific research institutions and generally reflected the ME research status. A possible reason could be that the ME research field is highly academic in nature and not currently economically feasible, thus enterprises and other institutions may currently eschew the field. The Medical University of Vienna and the National University of Singapore were major prominent organizations which had significant ME research outputs.

### 3.3 Journals

**FIGURE 6:**
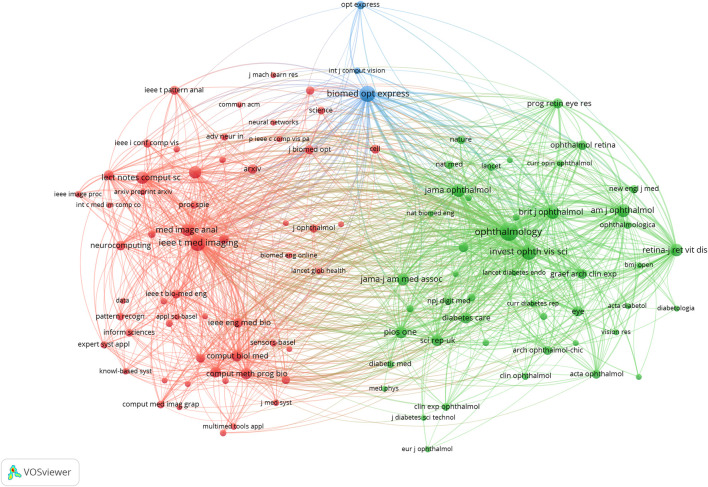
Network map showing how cited journals contributed to publications.

Across all academic fields, knowledge exchange in/between fields is often reflected in reference relationships between academic journals. Citing papers are knowledge frontiers, while referenced papers are knowledge bases. As indicated (Figure 6; Table 2), the major journals contributing to ME research included, Biomed Opt Express, Ieee T Med Imaging, Ophthalmology, Med Image Anal, and Invest Ophth Vis Sci—with high centrality, these were the most popular journals publishing ME research.

**TABLE 2:** Top ten macular edema artificial intelligence citations.

Rank	Source titles	Title of References	Count	Interpretation of findings
1	Jama-Journal Of The American Medical Association	Development and Validation of a Deep Learning Algorithm for Detection of Diabetic Retinopathy in Retinal Fundus Photographs	2,919	Detecting diabetic retinopathy (DR) using deep learning (DL)
2	Cell	Identifying Medical Diagnoses and Treatable Diseases by Image-Based Deep Learning	1,465	Using an artificial intelligence (AI) algorithm for retinal optical coherence tomography (OCT) image diagnoses
3	Nature Medicine	Clinically applicable deep learning for diagnosis and referral in retinal disease	952	Establishing a referral recommendation framework based on DL algorithms for retinal diseases which endanger vision
4	Investigative Ophthalmology and Visual Science	Improved Automated Detection of Diabetic Retinopathy on a Publicly Available Dataset Through Integration of Deep Learning	460	Using a convolutional network method to automatically detect DR when compared with other automated detection methods (IDx DR X2.1)
5	Biomedical Optics Express	Automatic segmentation of nine retinal layer boundaries in OCT images of non-exudative AMD patients using deep learning and graph search	306	A new framework automatically segmenting nine-layer boundaries in retinal OCT images
6	Biomedical Optics Express	ReLayNet: retinal layer and fluid segmentation of macular optical coherence tomography using fully convolutional networks	297	A Relay Net strategy to segment multiple retinal layers and delineate fluid pockets in OCT images
7	Progress In Retinal and Eye Research	Artificial intelligence in retina	278	Introducing AI to the retina
8	Ophthalmology	Fully Automated Detection and Quantification of Macular Fluid in OCT Using Deep Learning	233	A DL method which automatically detects and quantifies intra retinal cystic and subretinal fluid
9	Biomedical Optics Express	Deep-learning based, automated segmentation of macular edema in optical coherence tomography	181	A segmentation method based on DL and segmented intraretinal fluid
10	Progress in Retinal and Eye Research	Deep learning in ophthalmology: The technical and clinical considerations	171	Technologies and considerations are outlined for the construction of DL algorithms in ophthalmological/clinical settings

**FIGURE 7:**
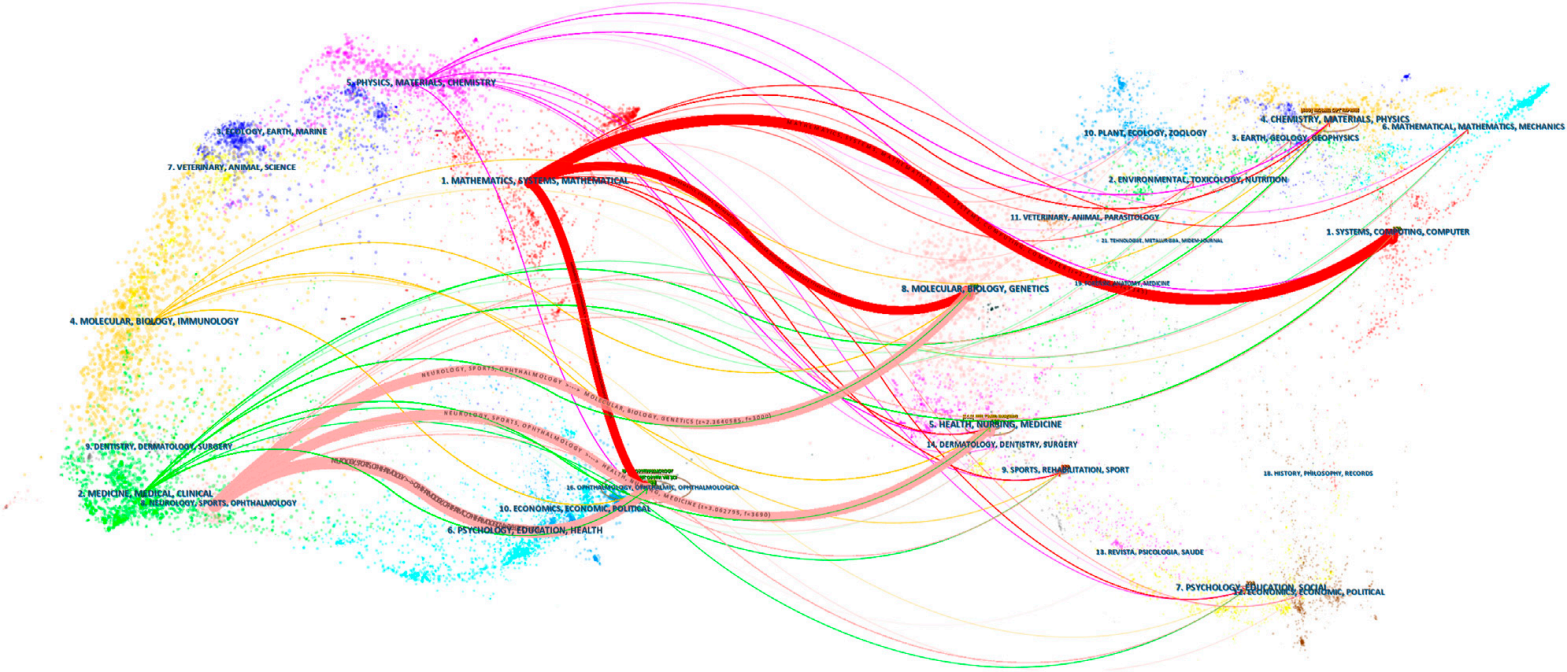
Dual-map overlay showing journal contributions to studies.

A journal dual-map overlay (Figure 7) showed citing (left) and cited (right) journals, while citation associations were indicated by colored lines—these investigations demonstrated that studies in computer/medicine/molecular journals were typically cited in ophthalmology/mathematics/clinical journals.

### 3.4 References

Reference analysis is an important index in bibliometric—typically, often-cited studies significantly impact certain research areas. Therefore, we used Citespace to cluster references from data to generate a reference clustering diagram (with a timeline) to analyze ME research.

**FIGURE 8:**
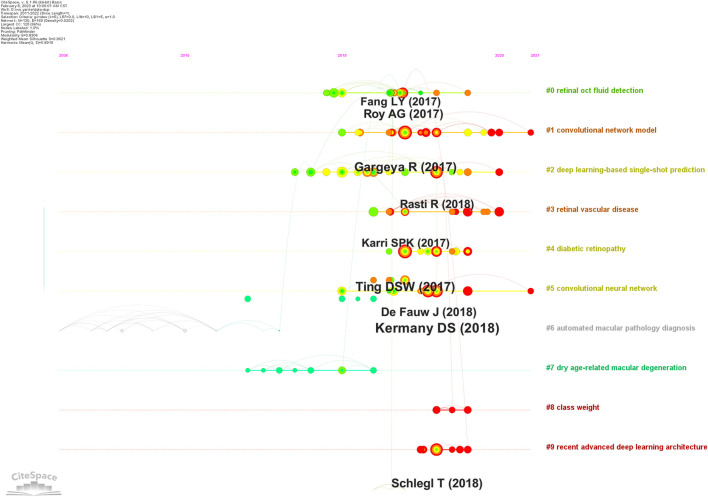
Reference co-citation map showing macular edema studies (2011–2022).

A co-citation reference network was used to assess the relevance of studies (Figure 8). Cluster setting: g-index K = 5 and #years/slice = 1. The modularity Q score was 0.8306 (>0.5), thus the network was adequately split into loosely coupled clusters. The weighted mean silhouette score was 0.9621 (>0.5), thus cluster homogeneity was reasonable.

Index items, as cluster markers, were extracted from studies. The largest clusters were cluster #0 “retinal oct fluid detection” (Lee et al., 2017; Venhuizen et al., 2018; Girish et al., 2019), cluster #1 “convolutional network model” (Gargeya and Leng, 2017; Porwal et al., 2020; Singh and Gorantla, 2020; Dai et al., 2021), cluster #2 “deep learning-based single-shot prediction” (Rasti et al., 2018; Das et al., 2019; Tsuji et al., 2020), cluster #3 “retinal vascular disease” (Karri et al., 2017; Ehlers et al., 2019; Figueiredo et al., 2020; Rasti et al., 2020), cluster #4 “diabetic retinopathy” (Ting et al., 2017; Raumviboonsuk et al., 2019; Ting et al., 2019), cluster #5 “convolutional neural network” (Kermany et al., 2018; Hwang et al., 2019), cluster #6 “automated macular pathology diagnosis” (Chang and Lin, 2011), cluster #7 “dry age-related macular degeneration” (Kafieh et al., 2013; Srinivasan et al., 2014a; Rathke et al., 2014; Karri et al., 2016), cluster #8 “class weight”(Wan et al., 2018; Li et al., 2019b; Huang et al., 2019), and cluster #9 “recent advanced deep learning architecture” (Schmidt-Erfurth et al., 2018; Bogunovic et al., 2019; Gu et al., 2019; Lu et al., 2019).

### 3.5 Keywords

**FIGURE 9:**
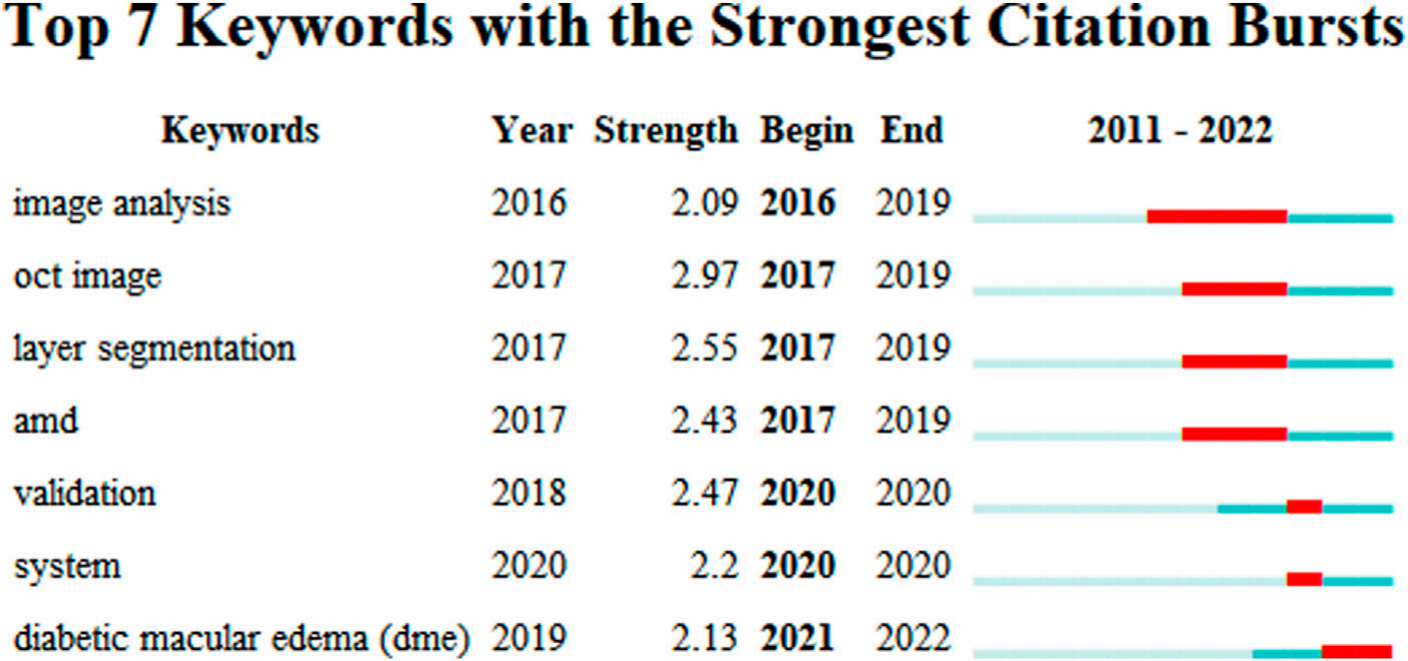
Keywords with the strongest citation bursts in macular edema studies (2011–2022).

Keyword analyses help summarize research themes and explore research hotspots and trends in a given field. The top 20 keywords from ME studies are shown (Table 3). Temporal trend/hotspot shifts (from seven keywords with the strongest citation burst) in 2016–2019; BKs were Image Analysis (2016–2019), OCT Imaging (2017–2019), Layer Segmentation (2017–2019), and Age Related Macular Degeneration (AMD) (2017–2019). BKs in 2020–2022 were validation (2020), system (2020), and the hotspot, Diabetic Macular Edema (DME) (2021–2022). (Figure 9).

**TABLE 3:** The top 20 keywords and associated strength data.

Rank	Keyword	Occurrence	Link strength	Rank	Keyword	Occurrence	Link strength
1	Optical coherence tomography	110	356	11	Diabetic-retinopathy	35	108
2	Diabetic macular edema	94	329	12	Automated detection	32	138
3	Deep learning	87	337	13	Images	32	106
4	Macular edema	75	226	14	Artificial intelligence	30	136
5	Diabetic retinopathy	62	225	15	Ranibizumab	30	86
6	Degeneration	57	208	16	Retina	28	120
7	Classification	52	196	17	Prevalence	26	85
8	Segmentation	50	182	18	Fluid	25	86
9	Retinopathy	48	212	19	Diseases	24	113
10	Validation	37	156	20	Machine learning	24	96

## 4 Discussion

### 4.1 General data

Between 2011 and 2022, 297 ME studies, conforming to inclusion/exclusion criteria and search terms, were identified. China generated the most studies (101, 34.007%), with the United States in second place (73, 24.579%). Two of the top ten institutions were in China. The most common ME publication journal, and the major contributor to ME research, was BIOMEDICAL OPTICS EXPRESS. The top study (cited 2,936 times) was by Gulshan et al. in JAMA-JOURNAL OF THE AMERICAN MEDICAL ASSOCIATION (Gulshan et al., 2016). The second top study (cited 1,474 times) was by Kermany et al. in CELL (Kermany et al., 2018).

### 4.2 Knowledge base

Previously DL-related technologies and associations with ME generated several major achievements. When co-cited references were clustered (Figure 8), key clustering nodes were used to identify knowledge bases in ME research: #0 “retinal oct fluid detection,” #1 “convolutional network model,” #2 “deep learning-based single-shot prediction,” #3 “retinal vascular disease,” #4 “diabetic retinopathy,” #5 “convolutional neural network,” #6 “automated macular pathology diagnosis,” #7 “dry age-related macular degeneration,” #8 “class weight,” and #9 “recent advanced deep learning architecture.” In the following sections, we outline knowledge bases according to different clusters.

#0 “Retinal OCT fluid detection”; Lee et al. (2017) generated a model formulated on encoding and decoding mechanism and outlined improved segmentation intraretinal fluid (IRF) methods, which showed good IRF segmentation results in OCT images. Roy et al. (2017) developed an AI approach (Relay Net) which segmented multiple retinal layers and generated fluid bag descriptions in OCT eye images. The model displayed excellent performance in object segmentation. Fundus dropsy can lead to ME. These methods were used to segment retinal fundus dropsy and automatically detected ME in OCT and affected segmented parts.
#1 “Convolutional network model”; Gargeya and Leng. (2017) designed a convolution network model to facilitate automatic DR recognition. Abramoff et al. (2016) compared this convolutional network with other automatic detection methods (IDx-DR X2.1) to automatically detect DR, mainly evaluating the analysis software that IDx-DR X2.1 runs on the server maintained and controlled by IDx. Porwal et al. (2020) generated a dataset for Indian populations with DR which provided normal retinal and typical DR structures at pixel levels. Image information was also provided for DR and DME severity, and facilitated image algorithm development and evaluations for early DR detection. DR is one of the most common diabetic microvascular complications. Retinal microvascular leakage and occlusions caused by chronic progressive diabetes causes different fundus diseases. DR is one of the main inducements of ME. Importantly, automated DR screening combined with a convolutional network model can effectively and economically prevent ME.
#2 “Deep learning-based single-shot prediction”; Rasti et al. (2018) used a DL-based single-shot prediction method (MCME) to predict macular OCT categories. The model performed category predictions based on minimum preprocessing requirements and helped to automatically classify macular OCT in clinical settings. The method by Srinivasan et al. (2014b) automatically detected diabetic ME and DARMD in OCT images. Using Histogram of Oriented Gradient descriptors and SVMs to classify spectral domain-OCT images, the method may be used to remotely diagnose some ophthalmic diseases.
#3 “Retinal vascular disease”; Karri et al. (2017) used a DL technique to identify DME or DARMD in retinal vascular diseases from OCT images. The strategy used transfer learning and the pre-trained GoogLeNet as a classification model to allow for faster convergence with less data. Li et al. (2019a) combined four classification models to automatically detect four retinal vascular diseases in OCT images: choroidal neovascularization, DME, DRUSEN, and NORMAL. The method had a classification accuracy = 0.973, which met or exceeded ophthalmologist expectations.
#4 “Diabetic retinopathy”; a DL system by Ting et al. (2017) was used to rapidly and accurately screen DR and related eye diseases. Abramoff et al. (2018) diagnostically evaluated an autonomous AI system (mtmDR) to automatically detect DR and DME; the approach improved early DR detection rates and reduced pain induced by vision loss and blindness. Thus, to some extent, these methods helped limit ME.
#5 “CNN” is one of the representative DL algorithms (Gu et al., 2018). Gulshan et al. (2016) developed a CNN algorithm to detect DR in retinal fundus images and detect referential diagnostic retinopathy. Kermany et al. (2018) formulated an effective transfer learning algorithm which processed medical images and identified key pathology traits in images. The algorithm was primarily used to analyze retinal OCT images, while combination with a CNN helped clinicians effectively diagnose ME.
#6 “Automated macular pathology diagnosis”; Chang and Lin (2011) introduced a software package for SVM algorithms—the LIBSVM library—which is one of the most widely used SVM software programs. The algorithm effectively supported automatic macular pathological diagnoses using SVM.
#7 “Dry age-related macular degeneration”; Chiu et al. (2015) designed a method to automatically segment diabetic ME in OCT images. The authors first estimated fluid and retinal layer positions using a classification method based on kernel regression, and then used classification estimates to accurately segment retinal layer boundaries using dynamic programming frameworks and graph theory. The method was the first to be validated, fully-automated, seven-layered, and fluid segmented for analyzing severe real-world DME images. Karri et al. (2016) generated a structured learning algorithm which enhanced layer specific-edge detection in OCT retinal images. Simultaneously, the algorithm identified layers and corresponding edges so that layer-specific edge computation were calculated to within 1 s.
#8 “Class weight”; Huang et al. (2019) formulated a layer-guided CNN to classify OCT retinal images. The method was divided into; 1), a segmentation network (ReLayNet) extracted segmentation maps from retinal layers, and 2), two disease related layers (RPE-BrM and ILM-RPE) were taken from layer segmentation graphs. The network may be applied to other retinal diseases (macular hole and macular telangiectasia) and also retinopathy detection and segmentation.
#9 “Recent advances in deep learning architecture”; Bogunovic et al. (2019) reviewed the standards and models used in retinal OCT fluid detection and segmentation, and showed that >50% of clinical teams selected UNet and its derivative model structure as a basic network architecture to segment OCT images. Schlegl et al. (2018) generated a DL strategy to automatically quantify and detect subretinal fluid (SRF) and intra-retinal cystic fluid (IRC). The method included a CNN with encoder/decoder architecture, which identified IRC and SRF. In the ME research field, codec structures (similar to UNet) have become popular DL network architecture approaches.

### 4.3 Frontiers and hotspots

Keywords typically highlight research ideas, while BKs reflect research frontiers and trends. Citespace captured BKs and identified ME research frontiers; e.g., DME in 2021–2022. We forecast these words will highlight future research frontiers ME research.

DME represents retinal thickening or hard exudative deposition caused by extracellular fluid accumulation in the optic disc diameter, in the macular fovea. OCT image are important tools for diagnosing diabetic macular disease, and AI-related methods for identifying and segmenting disease related ME diabetes in OCT image are key modalities for clinicians who treat and screen diseases and help reduce medical costs.

When AI correlation methods were used to assess OCT images, (Sunija et al., 2021; Nazir et al., 2021; Tayal et al., 2021; Wu et al., 2021; Atteia et al., 2021) used DL to automatically recognize ME-related lesions in OCT images. Similarly, He et al. provided accurate image support for doctors when diagnosing ME by layering retinas in images.

Sunija et al. (2021) used a lightweight DL algorithms to determine if patients had DME from OCT images. The algorithm network comprised six deep CNN layers and had accuracy and recall rates of 99.69% and 99.69%, respectively.

He Y. et al. (2021) formulated a unified framework for segmenting structured layer surfaces which generated continuous structured and smooth layer surfaces, with ordered topology, in an end-to-end DL strategy. DME was effectively observed by layering the retinal surface, and generating sub-pixel surface positions in single feed-forward propagation with full connection layers, thereby improving segmentation accuracy.

Nazir et al. (2021) proposed an automatic DR and DME screening method. The algorithm used DenseNet-100 as the basic CNN architecture and was greatly improved. The approach also extracted representative information from low-intensity/noisy images and accurately classified them.

The diagnostic method by Tayal et al. (2021) automatically detected DME and used three different CNN models (five, seven, and nine layer approaches) to classify and recognize four eye diseases. The strategy generated high F1 scores, accuracy and sensitivity outputs, and greatly reduced detection times.

Wu et al. (2021) developed a DL model to detect morphological DME patterns based on OCT images using a VGG-16 network strategy. The model was trained using ME manifestations in OCT images (diffused retinal thickening, cystoid ME, and serous retinal detachment) and greatly facilitated disease diagnostics.

Atteia et al. (2021) formulated a transfer-based stacked autoencoder neural network system, which used four standard pre-training depth networks to extract information from small input datasets. With a maximum classification accuracy = 96.8% and specificity = 95.5%, the approach allowed clinicians to automatically detect and diagnose DME.

## 5 Conclusion

We performed a bibliometric investigation of ME research related to DL, machine learning, FCN, CNN, RNN, and other AI fields. We identified the ME knowledge base, future trends, and current research hotspots. The knowledge base included: retinal OCT fluid detection, convolutional network models, DL-based single-shot predictions, retinal vascular disease, DR, CNNs, automated macular pathology diagnosis, DARMD, class weight, and recent advances in DL architecture. DME was also identified as a future research trend and Frontier. The current research focuses on disease classification in OCT images, segmentation and segmentation of disease regions based on OCT images.

Our study had some limitations; we only identified studies between 2011 and 2022, therefore, some research may have been missed, thus we possibly and inadvertently introduced publication bias into our investigation which impacted our conclusion.

## Data Availability

The datasets presented in this study can be found in online repositories. The names of the repository/repositories and accession number(s) can be found in the article/Supplementary Material.
